# Targeting EP2 Receptor for Drug Discovery: Strengths, Weaknesses, Opportunities, and Threats (SWOT) Analysis

**DOI:** 10.1021/acs.jmedchem.3c00655

**Published:** 2023-07-17

**Authors:** Thota Ganesh

**Affiliations:** Department of Pharmacology and Chemical Biology, Emory University School of Medicine, Atlanta, Georgia 30322, United States

## Abstract

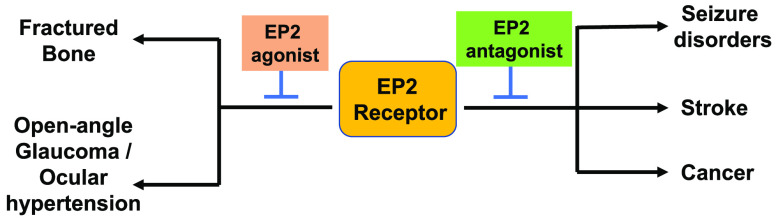

Cyclooxygenase-1 and -2 (COX1 and COX2) derived endogenous ligand prostaglandin-E_2_ (PGE_2_) triggers several physiological and pathological conditions. It mediates signaling through four G-protein coupled receptors, EP1, EP2, EP3, and EP4. Among these, EP2 is expressed throughout the body including the brain and uterus. The functional role of EP2 has been extensively studied using EP2 gene knockout mice, cellular models, and selective small molecule agonists and antagonists for this receptor. The efficacy data from in vitro and in vivo animal models indicate that EP2 receptor is a major proinflammatory mediator with deleterious functions in a variety of diseases suggesting a path forward for EP2 inhibitors as the next generation of selective anti-inflammatory and antiproliferative agents. Interestingly in certain diseases, EP2 action is beneficial; therefore, EP2 agonists seem to be clinically useful. Here, we highlight the strengths, weaknesses, opportunities, and potential threats (SWOT analysis) for targeting EP2 receptor for therapeutic development for a variety of unmet clinical needs.

## Introduction

Over the last two decades, the prostaglandin-E_2_ (PGE_2_) receptor EP2 subtype has gained tremendous attention. Using genetic knockout and pharmacological methods, the roles of EP2 have been delineated in various central nervous system (CNS) and peripheral disease models.^1^ EP2 is a Gα_s_-protein coupled receptor, which upon binding with endogenous ligand PGE_2_ activates adenylate cyclase resulting in the synthesis of cAMP which promotes intracellular signaling via protein kinase A (PKA) or exchange protein activated by cAMP (Epac).^2^ The PKA-mediated signaling is associated with neuroprotection and neuroplasticity, whereas Epac-signaling is associated with neuroinflammation and neurodegeneration in the central nervous system.^2^ Physiologically, the Gα_s_-coupled (cAMP) mediated signaling is also associated with smooth muscle relaxation; thus EP2 acts as muscle relaxant. In the periphery, EP2 receptor also promotes G-protein independent signaling through β-arrestin via c-Jun-N-terminal kinase (JNK) or extracellular signal regulated kinase (ERK) leading to cancer proliferation, tumorigenesis, and metastasis.^2^ Moreover, EP2 activation increases IL-23 expression that causes T-cells to differentiate to Th17 (from Th0) effectors, leading to chronic inflammation, and increased recruitment of neutrophils and macrophages to the injured site resulting in exacerbation of disease pathology.^3^

The structural homology (amino acid identity) between the EP2, EP1, EP3, and EP4 receptors, which share the common endogenous ligand PGE_2_ for their activation is limited to only 20–30%.^4^ Among these, EP4 is also coupled with Gα_s_-protein and mediates the cAMP signaling like EP2 receptor. Therefore, EP2 and EP4 have similar functional roles in several diseases. Interestingly, EP4 also plays an opposing functional role in several other diseases (see below). The EP1 is Gq-coupled and regulates phosphoinositide 3-kinases (PI3K) raising the cytosolic Ca^2+^ levels, whereas EP3 is G_i_-coupled and inhibits the adenylate cyclase resulting in lowering the levels of cAMP. In addition to EP receptors, EP2 receptor has structural similarity to extended family members of prostanoid receptors such as DP1 (44%), IP (40%), and FP and TP (20%).^4^ It is important to highlight that DP1 and IP receptors are coupled to Gα_s_-protein. DP1 receptor upon binding with endogenous ligand PGD_2_, and IP receptor upon binding with endogenous ligand PGI_2_ activate the adenylate cyclase resulting in synthesis of cAMP similar to EP2 receptor. This cAMP activity is involved in smooth muscle relaxation.^3^ Functionally, DP1 receptors play similar roles as EP2 depending on the disease phenotype, while the IP receptor role is mainly cardioprotective, meaning inhibition of IP receptor would lead to adverse effects on the cardiovascular system and other physiological functions.^5^

Recently, several studies have reported EP2 receptor as a key promoter of neuroinflammation in brain injury models and peripheral inflammatory diseases suggesting EP2 inhibition with small molecules would be therapeutically beneficial.^1^ Some studies also indicate that EP2 activation with agonist is therapeutically beneficial in healing the bone from a fracture,^6^ reducing intraocular pressure,^7^ and anti-inflammatory and bronchodilatory effects in the lung,^8^ reinforcing the idea that EP2 receptor is a novel therapeutic target for drug discovery for a variety of diseases. However, there are also some potential concerns about EP2 as a therapeutic target given its mechanism of action and “yin–yang” of the functional roles in physiological and disease-dependent pathological conditions. In this Perspective, we evaluate the *Strengths, Weaknesses, Opportunities, and Potential Threats (SWOT)* of the EP2 as a target for drug discovery, highlighting the strengths of proof-of-concept and efficacy studies in animal models, limitations associated with EP2 receptor targeting, and future studies needed to fulfill the knowledge gaps for clinical advancement of EP2 therapeutics. At the end, we also briefly highlight SWOT analysis of the currently available lead EP2 antagonists for preclinical development and clinical trials.

## Strengths

The goal of this Perspective is not to review all the proof-of-concept studies reported so far in various disease models exploring EP2 involvement, for which the reader is directed to recently published review articles.^1−3^ However, this Perspective’s purpose is to highlight the strengths and weaknesses of targeting EP2 receptor highlighting the opportunities and threats with studies that were conducted with scientific rigor using in vitro and in vivo models, and where both genetic and pharmacological approaches provide cohesively strong support as described below.

### Proof-of-Concept Studies in Central Nervous System Models

A recent study by Minhas et al. (2021)^9^ shows that EP2 receptor expression is higher in aged immune cells (human and mouse macrophages) than in young cells. Aged myeloid cells (microglia and macrophages) heavily depend on balanced glucose levels. EP2 receptor activation in aged microglia and macrophages promotes a microenvironment that converts glucose into glycogen, reducing the glucose flux and mitochondrial respiration and creating an energy deficient state that drives a malign inflammatory state.^9^ In aged mice, conditional deletion of EP2 from myeloid cells or treatment with EP2 antagonists rejuvenates cellular bioenergetics, systemic and brain inflammatory states, synaptic plasticity, and spatial memory, and blocking peripheral myeloid cell EP2 signaling restores cognition in aged mice, suggesting a role for EP2 receptor signaling in promoting the youthfulness of immune functions.^9^

Microglia (resident macrophages in the brain) perform critical functions such as clearing misfolded proteins and invading pathogens and balance trophic factors that maintain normal neuronal function. In the Alzheimer’s disease (AD) brain, these beneficial functions of microglia are impaired resulting in enhanced synaptic and neuronal loss. EP2 engagement in microglia suppresses the beneficial homeostatic functions of microglia, as a result the removal (or phagocytosis) of amyloid-β (Aβ) plaques is inefficient. A study by Johansson et al. (2015)^10^ showed that conditional deletion of EP2 in microglia in a mouse model of AD restores chemotaxis, Aβ-clearance, regulation of inflammatory milieu, and regeneration of trophic factors, leading to prevention of loss of synaptic proteins and cognitive deficits. Interestingly, ablation of microglial EP2 signaling improved spatial memory and increased presynaptic proteins in the APP-PS1 mouse model of AD.^10^ Along the lines of these findings, earlier reports indicate that microglia isolated from EP2 global knockout mice show enhanced phagocytosis of Aβ,^11^ and pharmacological antagonism of EP2 receptor with a small molecule inhibitor (C52, Figure 2) enhances peritoneal macrophage mediated phagocytosis of Aβ_42_,^12^ a key driver of AD. In an emerging study from our laboratory, chronic treatment of EP2 antagonist in 5×FAD mice starting at the prodromal stage (starting from 3 months of age until they are 5 months) in drinking water showed reduced inflammatory mediators and gliosis in the cortex,^13^ and this effect was only found in a two-hit model of 5×FAD (genetic 5×FAD mice were subjected to chronic but mild LPS treatment for 2 months).^13^ These studies conclude that EP2 is deleterious in age-related and AD conditions; therefore small molecule inhibitors (EP2 antagonists) must be advanced for the treatment of age-related and Alzheimer’s diseases.

**Figure 1:**
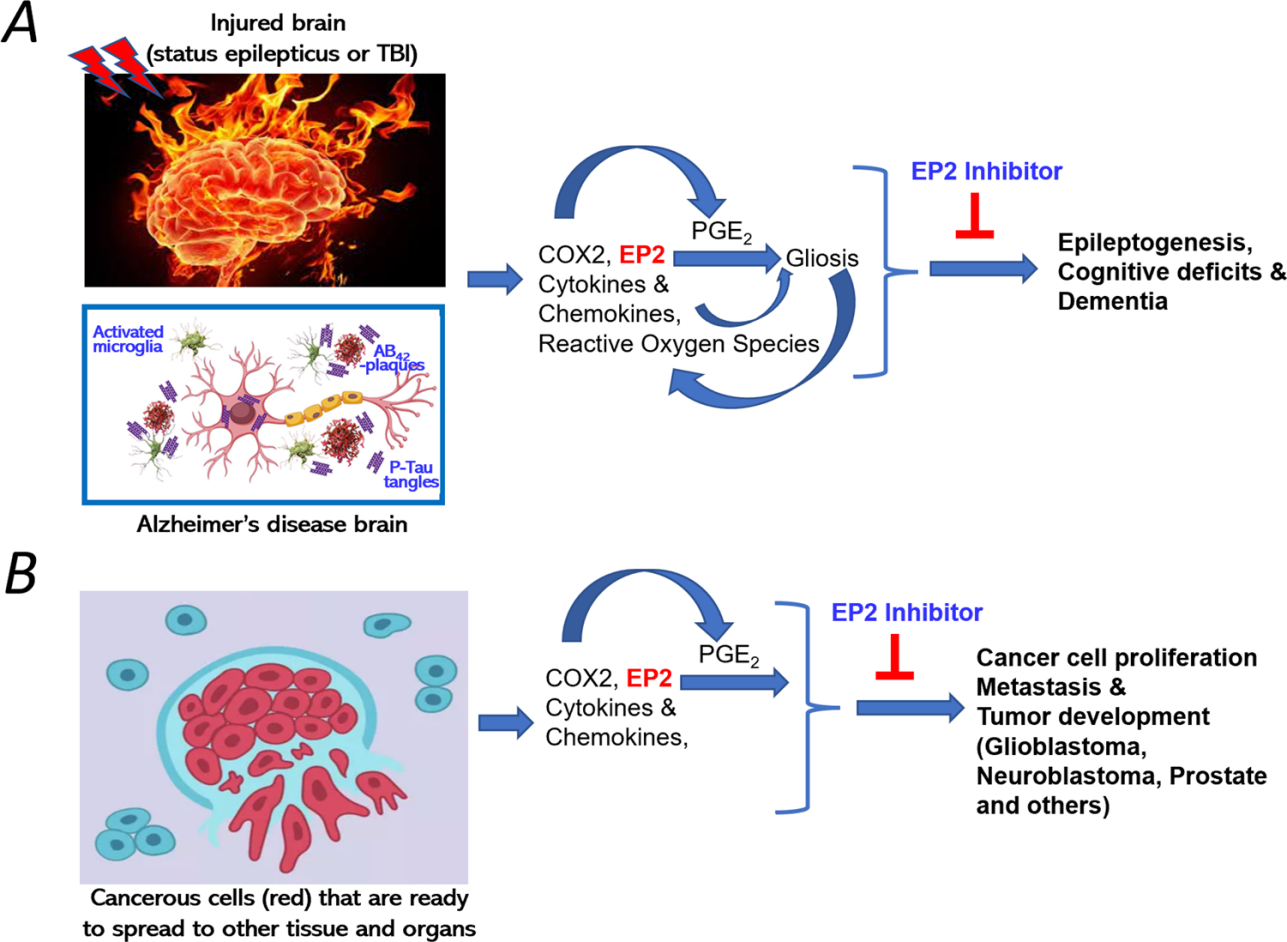
(A) Illustration of EP2 involvement in gliosis and subsequent neurodegenerative pathologies such as epilepsy, memory and cognitive deficits, and Alzheimer’s disease type dementia. (B) EP2 involvement in inflammation driven cancer proliferation, metastasis, and tumor development. EP2 inhibition with a small molecule antagonist should be therapeutically beneficial.

**Figure 2:**
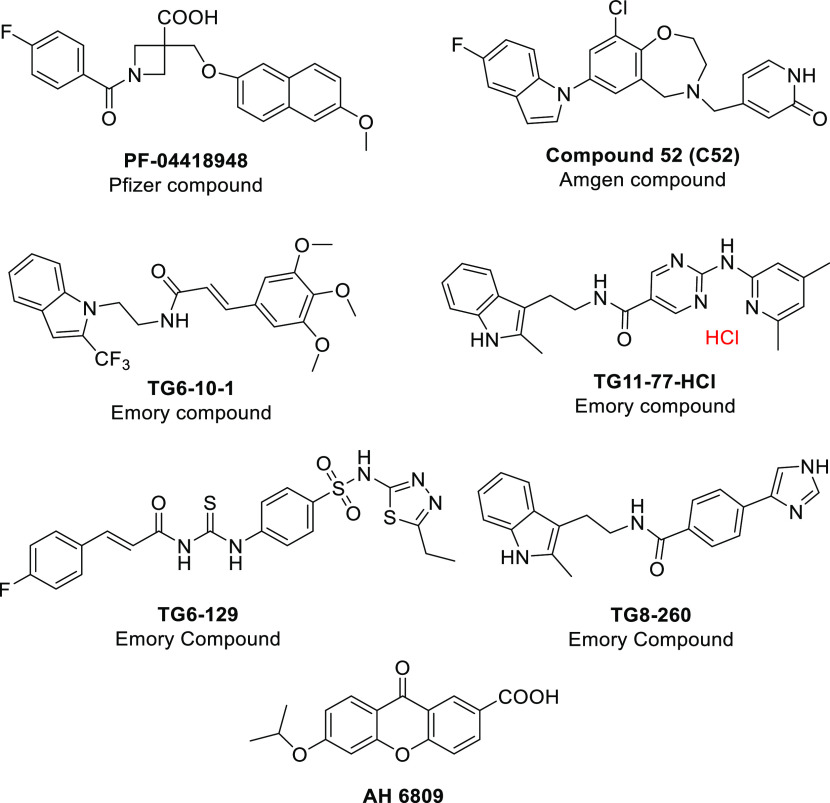
EP2 antagonists so far tested in animal proof-of-concept studies. Among these, AH 6809 is a dual antagonist of EP1 and EP2 receptors with equal potency.

Microglia (myeloid cells) play a key role in several other neurodegenerative disease pathologies. Acute brain injuries due to status epilepticus (SE) and traumatic brain injury (TBI) create massive neuroinflammation (microgliosis, astrogliosis, and induction of cytokines, chemokines, and cyclooxygenase-2 (COX2)) in the brain,^2^ which will exacerbate secondary neurodegenerative pathology leading to cognitive and behavioral deficits. Our laboratory investigated the role of EP2 in SE models (mouse and rat),^14−18^ and fluid percussion (TBI) injury in rats (Figure 1).^19^ Very recently, we (Varvel et al, 2021)^20^ have shown that conditional ablation of EP2 from blood monocytes or systemic EP2 antagonism with EP2 antagonist (TG6-10-1, Figure 2) blocks monocyte entry to the mouse brain after SE, and post-SE treatment with an EP2 antagonist remarkably prevents the breakdown of the blood–brain barrier (BBB), up-regulation of inflammatory markers, and neurodegeneration in the hippocampus of mice 3 days postrecovery from pilocarpine-induced SE. We have also reported that treatment with another EP2 antagonist, TG11-77, reduces not only microgliosis in the hippocampus but also the cognitive deficits determined 8–18 days after recovery from pilocarpine-induced SE in mice.^21^ In this later study, TG11-77 did not provide any neuroprotection in the hippocampus after SE injury, providing a rationale for the hypothesis that anti-inflammatory efficacy is sufficient to modulate function of microglia to enhance cognitive function. These studies in conjunction with several others in rat and mouse models show that EP2 antagonism reduces the delayed mortality, expression of neuroinflammatory mediators, and neurodegeneration in the hippocampus, repairs the BBB, and prevents peripheral myeloid cell entry into the brain days following SE. This evidence strongly indicates that EP2 receptor is a suitable drug target for the development of therapeutic agents for treatment of the consequences of SE injury (event) and the ensuing cognitive deficits and potentially for the prevention of epileptogenesis after traumatic brain injuries.^19^

Cognitive impairments are common among the survivors of stroke, sepsis, and respiratory syncytial virus (RSV) and other severe acute respiratory syndrome (SARS) virus infections. Lipopolysaccharide (LPS) has been shown to mimic sepsis phenotypes. Systemic exposure to LPS induces massive neuroinflammatory conditions and cognitive deficits after recovery as recently shown by Jiang et al. (2020).^22^ In this study, EP2 antagonist TG6-10-1 treatment, attenuated not only the massively up-regulated neuroinflammation (microgliosis and inflammatory markers such as IL-6, IL-1β, TNFα, COX2, iNOS) in the hippocampus and loss of synaptic proteins (PSD-95) but also depressive symptoms and memory impairment in mice. Since this LPS model did not (or does not) present any neurodegeneration phenotype in the hippocampus, we have not been able to confirm model independent neuroprotective efficacy by the EP2 antagonist in this model. Nonetheless, these results support the hypothesis that neuroinflammation alone is sufficient to cause cognitive deficits in rodents; EP2 antagonists with and without neuroprotective activity will offer cognitive improvements,^21^ providing support toward clinical advancement of EP2 antagonist for several CNS diseases such as SE, AD, and aging-related illnesses. Please see Table 1 for an overview of efficacy results described in this section.

**Table 1:** Overview of Selected EP2 Receptor Antagonists and Their Efficacy in Various Rodent Models

EP2 antagonist	brain-permeable?	rodent model	efficacy markers	ref
PF-04418948	no	rat	reversed butaprost-induced cutaneous flow in rats	(32)
PF-04418948	no	endometriosis mouse model	reversed mechanical allodynia tested on the abdomen and hind-paw	(33)
PF-04418948	no	naive aged mice	reversed age-associated hippocampal memory deficits and restored the long-term potentiation; reduced pro-inflammatory factors in the blood and also in the hippocampus	(9)
C52	yes	naive aged mice	reversed age-associated spatial memory deficits by novel object recognition (NOR) and Barnes maze tests; reversed age associated inflammation in the plasma and hippocampus	(9)
C52	yes	ischemia (MCAo) mouse model	reduced infarct volume; increased neurological score	(23)
TG6-10-1	yes	ischemia (MCAo) mouse model	reduced infarct volume; down-regulated proinflammatory cytokines in the brain	(24)
TG6-10-1	yes	endometriosis mouse model	reversed mechanical allodynia on the abdomen; trend in decreasing mechanical allodynia on the hind-paw	(33)
TG6-10-1	yes	pilocarpine-induced status epilepticus (SE) mouse	reduced inflammation and gliosis in the hippocampus; reduced neurodegeneration in the hippocampus; protected blood–brain barrier; reduced delated mortality and accelerated recovery from SE	(14)
TG6-10-1	yes	diisopropyl fluorophosphate (DFP)-induced SE rat	blunted inflammatory cytokine burst and microgliosis in the hippocampus; blunted neurodegeneration in the hippocampus; accelerated recovery from SE; blunted memory impairments by NOR test	(17, 18)
TG6-10-1	yes	kainate-induced SE model	reduced hippocampal inflammatory cytokines; reduced neurodegeneration in CA3 region of hippocampus; blunted blood monocyte entry into the brain; prevented breakdown of blood–brain barrier; accelerated recovery from SE	(20)
TG6-10-1	yes	LPS-induced sepsis-associated encephalopathy (SAE)	blunted inflammatory cytokines and gliosis in the hippocampus and prevented the loss of synaptic proteins; accelerated body weight recovery; blunted depression-like behavior by sucrose preference test; blunted memory loss by NOR tests	(22)
TG6-10-1	yes	glioblastoma mouse models	reduced growth of subcutaneous tumors in athymic nude mice; suppressed intracranial tumors in xenograft model; increased survival of mice that harbored intracranial tumors	(34)
TG8-260	no	pilocarpine-induced SE rats	blunted inflammatory cytokines and gliosis in the hippocampus; no effect on neurodegeneration and blood–brain barrier breakdown	(16)
TG11-77	yes	pilocarpine-induced mouse SE model	blunted inflammatory cytokines in the hippocampus; reduced microgliosis in the hippocampus; eliminated memory deficits determined by Y-maze; no effect on neurodegeneration and blood–brain barrier breakdown	(21)
TG11-77	yes	two-hit 5×FAD mouse model of Alzheimer’s disease	blunted inflammatory cytokines only in females; blunted glial proteins only in females	(13)
TG6-129	no	neuroblastoma mouse models	suppressed the growth of neuroblastoma xenografts in nude mice; suppressed syngeneic neuroblastoma in immunocompetent mice	(35)

The above studies indicate that EP2 receptor suppresses beneficial functions of microglia and blocking myeloid EP2 signaling reduces pathology in inflammatory neurodegenerative models. Another recent study by Liu et al. (2019)^23^ reports that in mouse model of stroke, in which the initial ischemic event was followed by extended poststroke inflammatory response, EP2 knockdown from myeloid cells (Cd11bCre:EP2^lox/lox^ mice) attenuated the infiltration of macrophages (Cd11^+^CD45^hi^) and neutrophils (CD45^+^Ly6G^hi^). Inducible global deletion of EP2 receptor in adult mice (ROSACreER;EP2^lox/lox^) also reduced the infiltration of myeloid cells to the brain and stroke severity in these mice.^23^ EP2 expression is highly induced in neurons after ischemic injury, postnatal removal of neuronal EP2 in mice also reduced cerebral ischemic injury (infarct volume) in a middle cerebral artery occlusion (MCAo) stroke model, suggesting that EP2, irrespective of its cell origin, is involved in inflammatory brain damage and inhibition of EP2 signaling is protective after ischemic stroke events.^23^ Furthermore, these findings were reproduced with a pharmacological treatment of brain permeable EP2 antagonist (C52, Figure 2) 4.5 and 24 h after MCAo injury to mice, where this antagonist reduced the infarct volume and improved the neurological score consistent with results found in ROSACreER;EP2^lox/lox^ mice.^23^ These results were recapitulated by a similar study using another brain-penetrant EP2 antagonist, TG6-10-1, which showed decreased neurological deficits and infarct volumes as well as down-regulated inflammatory cytokines in the brain in a transient (MCAo) mouse model ischemia^24^ suggesting a novel strategy and strong rationale to develop therapeutic agents for treatment of stroke consequences by targeting EP2 receptor with small molecule antagonists.

It has been shown that EP2 deletion (global knockout) reduces Aβ-load and oxidative stress in a mouse model of AD^25^ extends the survival of mice and improves the motor strengths in an ALS model,^26^ and reduces neurotoxicity in a model of Parkinson’s disease.^27^ Several other studies showed that the PGE_2_/EP2 axis activates several innate immune pathways.^28−30^ Multiple sclerosis is an inflammatory autoimmune disorder of the CNS. COX2, mPGES-1, and EP2 expression are elevated in patients with multiple sclerosis. COX2 deletion or COX2 inhibition by celecoxib or EP2 inhibition by a dual EP2/EP1 antagonist AH6809 (Figure 2) reduced oligodendrocyte apoptosis, degree of demyelination and motor dysfunction in a cuprizone-induced model of multiple sclerosis.^31^ It is very important to note that several of these central nervous system disorders are associated with cognitive impairment and motor disabilities. Given our consolidated findings that suggest that EP2 driven neuroinflammation is strongly linked to cognitive and memory impairments and the fact that systemic administration of EP2 antagonist has attenuated cognitive and memory impairments and improved neurologic score, we foresee that EP2 receptor must be explored as a drug target for several of these neurologic diseases (Table 1).

### Proof-of-Concept Studies in Cancer Models

COX2 is highly expressed in a variety of cancers and exacerbates tumor aggressiveness through generation of precursor PGH_2_ for the synthesis of PGE_2_.^36^ Microsomal prostaglandin E synthase-1 (mPGES-1), the enzyme responsible for the last step of synthesis of PGE_2_ (from PGH_2_), is also highly induced in a variety of tumors.^37,38^ EP2 receptor activation (by PGE_2_) is associated with amplification of inflammation in the tumor microenvironment via induction of tumor promoting cytokines, chemokines, and growth factors.^2^ There is a positive correlation between COX2, mPGES-1, and EP2 receptor expression and inflammatory mediators that promote tumor proliferation, survival, migration, invasion, angiogenesis, and immune evasion in human gliomas.^34^ The study from Qiu et al. (2019)^34^ indicates that EP2 activation drives human glioma cell (GBM) proliferation and invasion in cell culture models in vitro that overexpress COX2 (LN229, and SF767) and overproduce PGE_2_. An EP2 antagonist, TG6-10-1, blocked the proliferation and invasion of these GBM cells, promoting apoptosis and cell cycle arrest. Moreover, in athymic nude mice that were inoculated subcutaneously with COX2 overexpressing SF767 cells, oral treatment with an EP2 antagonist for 4 weeks, significantly reduced the growth of subcutaneous tumors formed by SF767 cells, in which the average tumor burden (weight) was reduced by 63%. Additional experiments in this study indicate that glioblastomas typically display angiogenesis hallmarks (determined by platelet endothelial cell adhesion molecule 1 or CD31), which were increased by COX2 overexpression and then decreased by EP2 antagonist TG6-10-1 treatment indicating the role of EP2 in COX2 driven angiogenesis of gliomas. Furthermore, 4 weeks of EP2 antagonist treatment also suppressed orthotopic malignant gliomas from intracranially injected luciferase labeled LN229 glioblastoma cells in nude mice.^34^ These results are compelling to promote EP2 inhibitors for treatment of glioblastoma multiforme when the compound TG6-10-1 or any other EP2 antagonist meets the requisite ADMET characteristics for clinical development and clinical use.

**Figure 3:**
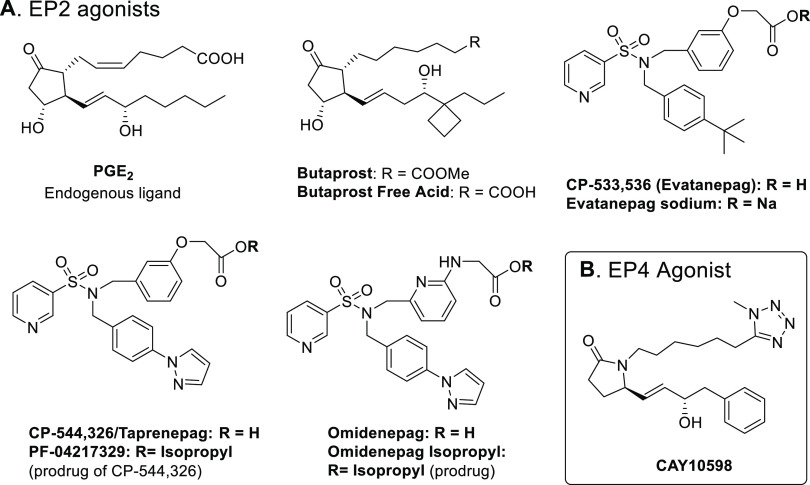
EP2 and EP4 agonists used in the described studies.

The expression of EP2 receptor is very high in high-risk neuroblastoma (NB) in comparison to other PGE2 receptors. The expression of EP2 is elevated among the nonsurviving patients compared to surviving NB patients, indicating that EP2 expression is coupled to poor survival of NB patients.^35^ Similarly, EP2 receptor is expressed at higher levels in NB patients with oncogenic MYCN gene amplification compared to NB patients with MYCN normal status suggesting a strong link to EP2 receptor in NB. MYCN amplification is the best characterized genetic marker of high risk and chemoresistance in NB.^39^ Interestingly, the other PGE_2_ receptors (EP1, EP3, and EP4) showed inverse correlation with MYCN in NB.^39^ Like in the glioblastoma study (above), the expression of EP2 was correlated with several tumor promoting cytokines, chemokines, growth factors, and receptors including anaplastic lymphoma kinase receptor (ALK), brain derived neurotrophic factor (BDNF), chemokine ligand-2 (CCL_2_), chemokine receptor-2 (CCR2), colony-stimulating factor-1 receptor (CSF1R), epidermal growth factor receptor (EGFR), and others.^35^ When NB cell lines with various risk factors (11q deletion (SK-N-AS), ALK mutation, MYCN amplification, P53 dysfunction, or KRAS mutation) were treated with EP2 agonists (PGE_2_ or butaprost, Figure 3A), but not EP4 agonist (CAY10598, Figure 3B), they displayed induction of cAMP, and this cAMP induction was similar to the activity of an adenylyl cyclase activator forskolin, suggesting that EP2 is involved via a Gα_s_-coupled mechanism in these cell lines. In a key in vivo experiment to determine EP2 involvement in tumorigenesis, 11q deleted NB cells (SK-N-AS), subjected to EP2 deletion by CRISPR–Cas9, or wild-type SK-N-AS cells were inoculated to athymic nude mice to generate high risk tumors with 11q deletion. The results show that tumors generated by EP2 deleted NB cells were significantly smaller in volume than the tumors generated by wild-type NB cells. Other than tumor volume, the mice that were given EP2 deleted NB cells were healthier overall, suggesting that EP2 is required for human high-risk NB cells to develop tumors. These findings were confirmed by multiple approaches including conditional deletion of EP2 and, importantly, by pharmacological treatment with EP2 antagonist TG6-129 (Figure 2 and see Table 1), for 18 days in a SK-N-AS NB inoculated mouse xenograft model. It is interesting to note that systemic treatment of TG6-129 substantially decreased the proliferation of tumors formed by SK-N-AS cells in a dose-dependent manner, about 25% reduction with a 10 mg/kg dose and 55% reduction with a 20 mg/kg dose for 18 days of once daily treatment.^35^ These studies strengthen the clinical development of small molecule EP2 antagonists and the EP2 receptor as a novel therapeutic target for a variety of medically untreated cancers.

### Proof-of-Concept Studies in an Endometriosis Disease Model

COX2 is up-regulated in endometriotic tissue and eutopic endometrial tissue, which synthesize a high level of PGE_2_ contributing to pathogenesis and exacerbation of endometriosis disease.^40,41^ Inhibition of COX2 decreases survival, migration, and invasion of endometriotic cells that are associated with decreased PGE_2_.^41^ There is a positive correlation between endometriosis induced vaginal hyperalgesia and the peritoneal fluid levels of PGE_2_.^33,42^ Moreover, EP2 receptor expression is also very high in the uterus,^20^ stromal cells in lesions, and mesothelial cells in the peritoneum.^33^ Based on this COX2/PGE_2_/EP2 signaling, Greaves et al. (2017)^33^ tested the role of EP2 in a mouse model of endometriosis monitoring the endometriosis pain mediated behaviors (licking and exploratory activities) and mechanical withdrawal from von Frey filament test. Treatment with EP2 antagonists, TG6-10-1 or PF-04418948 (Figure 2) (10 mg/kg dose), resulted in statistically significant reversal of mechanical allodynia on the abdomen and hind-paw tests (see Table 1). Interestingly, oral administration of PF-04418948 displayed time-dependent effects on mechanical withdrawal response in mice with endometriosis, with the withdrawal threshold significantly lowered in both abdomen and hind-paw tests of endometriosis mice compared with the naive controls.^33^ Although these results provide a strong impetus to advance an EP2 antagonist toward treatment of debilitating and medically unaddressed endometriosis disease, the detailed molecular and phenotypic changes in the endometrium are not investigated in this study to link it to the behavioral benefits. Moreover, the behavioral study was conducted with only *n* = 5 animals in each group; therefore, additional work with a larger number of animals is needed to enhance the strength of this work before the advancement of the EP2 target for endometriosis therapy.

## Weaknesses

Earlier studies to delineate the function of EP2 receptor in models of stroke, AD, SE, PD, ALS, and innate immunity were mostly carried out using EP2 global knockout (EP^–/–^) mice and in some cases using EP2 agonists (PGE_2_, butaprost, and CP-533,536, Figure 3), because of the lack of EP2 antagonists until 2011–2012 when highly characterized and selective EP2 antagonists were made available from Pfizer^32^ and Emory University^43^ laboratories (Figure 2). Subsequently, Amgen also reported a new class of EP2 antagonists in 2015 for investigation with in vitro and in vivo models.^12^

In a few studies, the results from EP2 global knockout mice are discordant with pharmacological inhibition or conditional deletion of EP2 from adult mice. For example, in a MCAo model of stroke, EP2 global deletion increased cerebral injury, with mice exhibiting impaired learning and memory,^44^ whereas conditional deletion of EP2 in microglia or neurons of adult mice attenuated the cerebral injury, with the mice exhibiting normal learning and memory phenotypes.^23^ Studies like these may create a perplexing view among the pharmaceutical community for discovery and advancement of any therapeutic agent to clinical trials for this disease. However, careful review of the confounding effects of EP2 at the developmental stage (pre- and postnatal period) versus adult stage, as shown by Liu^23^ offers a path forward. Several in vitro studies employing embryonic cortical or hippocampal neurons or hippocampal slice cultures from early postnatal brain suggest that EP2 is neuroprotective when pharmacologically activated with EP2 agonists or allosteric potentiators.^44−46^ These studies were conducted with insulting agents, glutamate, NMDA, and/or oxygen-glucose deprivation. However, given the recent assessment of EP2 expression in neurons of the adult brain, which is low in comparison to embryonic neurons, those in vitro culture results need to be carefully interpreted prior to comparing with in vivo efficacy studies with EP2 deletion and pharmacological approaches.

One of the key homeostatic functions of activated microglia is phagocytosis to clear debris in the brain. An in vitro study using microglia isolated from EP2 deleted mice (P1–P3 neonates) showed enhanced phagocytosis of Aβ-peptides from AD brain sections, compared to wild-type microglia.^11^ However, when BV2 microglia cells that overexpress human EP2 receptors are used for phagocytosis of fluorescent latex microspheres, the presence of EP2 but not the activation or inhibition of EP2 (by selective EP2 agonist or antagonist) caused an effect on the cells to phagocytose these latex microspheres.^47^ There are two main differences in these studies; we are comparing the results of primary microglia activity versus a transformed microglia like cell line (BV2) which may have confounding differences.^47^ Moreover, the subject of phagocytosis is different among the two studies (Aβ peptide vs fluorescent microspheres). Therefore, these studies must be interpreted independently and must not be compared to one other to draw conclusions on the role of EP2 in phagocytosis in vivo. Nonetheless, several other in vitro studies using primary microglia and peritoneal macrophages are consistent with an antiphagocytic role of EP2, where EP2 activation by PGE_2_ suppressed macrophage mediated phagocytosis of anti-CD36,^48^ which was reversed by treatment with a nonselective EP2/EP1 antagonist, AH 6809 (Figure 2). Nagano et al. reported that EP2 receptor activation with PGE_2_ dose-dependently reduced rat primary microglia mediated phagocytosis of amyloid-β_42_, which was reversed by treatment with AH 6809.^49^ Similarly, mouse peritoneal macrophage cells (IC21, ATCC TIB-186) when treated with EP2 antagonist, C52 (Figure 2), showed dose-dependent increase in phagocytosis of Aβ-plaques present in brain slices from 18 month old Tg2576 mice (AD).^12^ This effect of C52 (1 μM) was similar to that caused by 10 μg/mL Aβ-antibody in the same ex vivo assay, suggesting that EP2 expressed in myeloid cells is strongly associated with phagocytosis and it can be modulated with activation and inhibition strategies.

Similarly, several in vitro studies from our laboratory using primary rat microglia or mouse BV2-microglia cells overexpressing human EP2 receptors indicate that EP2 activation with an agonist results in up-regulation of several proinflammatory cytokines and chemokines (IL-1β, IL-6 and CCl2) and down-regulation of cytokine TNF^47,50,51^ suggesting that EP2 receptor has a mixed impact on proinflammatory gene expression. Pretreatment of these cultures with selective EP2 antagonists reverse inflammatory mediators expression (i.e., down-regulation of IL-1β, IL-6 and CCl2 and up-regulation of TNF) suggesting that EP2 antagonism is not completely a one-sided anti-inflammatory.^47,50,51^ This observation is discordant with findings from in vivo studies, where treatment with EP2 antagonist decreased the levels of IL-1β, IL-6, and CCl2 and TNF in 3 models of SE in two species (rat and mice).^2,14−17,21^ The in vitro up-regulation of TNF by EP2 antagonism can be explained by the finding that EP2 driven cAMP inhibits the release of TNF from the microglia or BV2 cells; therefore EP2 agonist decreases the levels of TNF, whereas EP2 antagonist interrupts this TNF blocking effect by the EP2/cAMP resulting in release of it, therefore we see EP2 antagonism increasing the TNF.^52,53^ However, this mechanism is occurring only in cell culture studies in vitro. In vivo (in SE model studies), EP2 typically increases the TNF levels and EP2 antagonist decreases them, consistent with anti-inflammatory properties for EP2 antagonist.

Moreover, we recently reported that EP2 is induced in rat microglia upon insult with LPS/IL-13, and the prolonged activation of EP2 with an agonist induces rat microglia death which can be prevented by treatment with EP2 antagonists.^54^ Cell death mediated by EP2 involves the activation of caspase-1 and -3 as well as generation of reactive oxygen species (ROS) promoting either pyroptosis or apoptosis mechanisms. In this study, microglia upon LPS/IL-13 treatment become swollen and round compared with the elongated form in resting condition, the activation of microglia EP2 with butaprost change the morphology of microglia causing them to shrink suggesting apoptosis. Whether EP2 receptor activation really causes microglia death in vivo is not clear. But in several studies,^10,55^ the inflammatory states of microglia are modulated by the presence and activation of EP2 receptor toward a maladaptive immune state. Therefore, these in vitro results must be interpreted individually and must not be compared with in vivo results to draw conclusions.

Global EP2 deletion is detrimental to mice. One study showed that EP2 deletion decreases reproduction rate, reduces litter size, and significantly elevates blood pressure in mice when they are on a high-salt diet compared with regular diet.^56^ Another study reported that EP2 global deletion causes salt-sensitive hypertension and reduced fertility.^57^ These two studies, published 20 years ago, had created a stumbling block for the development of EP2 antagonists that now show beneficial effects in several animal disease models as described in the Strengths section. These results obviously raise potential weaknesses, and they could even be threats if they are replicated by pharmacological EP2 antagonism. To address these weaknesses, we have recently conducted a study using two different EP2 antagonists, TG6-10-1 and TG11-77 (Figure 2). TG6-10-1 was administered by acute dosing in mice and rats, and TG11-77 was dosed by chronic oral dosing via drinking water. The rodents were subjected to regular or high-salt diets when they were on treatment with these two EP2 antagonists. We measured the systolic and diastolic blood pressure, heart rate, and respiratory function in mice and rats. Regardless of the diet in mice, these two antagonists did not cause any of the adverse phenotypes that were found in mice with EP2 global deletion.^58^ The discordance of the results from EP2 gene knockout and pharmacological antagonism can be attributed to the role of EP2 in development at prenatal and postnatal stages. Moreover, in the adult stage, the EP2 receptor seems to carry a majority of COX2/PGE_2_ driven inflammatory signaling. Therefore, the adverse phenotypes found in EP2 deleted mice must be interpreted as weaknesses rather than real threats for advancing the EP2 receptor as a therapeutic target with small molecule EP2 antagonists.

It has been very well-known that PGE_2_, via G protein-coupled signaling, is involved not only in inflammation but also in bone-formation and bone-healing, embryo implantation, induction of labor, and vasodilation indicating a “yin–yang” nature of PGE_2_ signaling depending on the injury and the disease.^2^ Endogenous PGE_2_ expression increased after bone fractures, and administration of PGE_2_ also stimulated bone formation in animal models.^59−61^ Both EP2 and EP4 receptors expressed in bone cells and marrow stromal cells are shown to play an important role in bone formation and resorption^62^ determined by using mice with either EP2 or EP4 knockout and the selective agonists of these two receptors. A selective EP2 agonist CP-533,536 (Figure 3) directly injected into bone marrow healed the fractured bone in rat and canine models.^6,63^ Pfizer has promoted this agonist for human clinical trials to examine efficacy, safety, and tolerability in subjects with closed fracture of the tibial shaft (https://clinicaltrials.gov/ct2/show/NCT00533377). Although clinical study results are not published to conclude the clinical utility of this agonist and clinical proof-of-evidence for EP2 agonism for fractured bones, the in vivo results from multiple models provide a strong rationale for EP2 agonists for local bone augmentation, bone repair, and healing.^6,63^ Similar beneficial effects were also found with use of selective EP4 agonists in these bone-fracture and bone-repair models,^64,65^ suggesting both receptors are involved in the bone repair and healing process. Similarly, Pfizer also promoted an EP2 agonist CP-544,326 (PF-04217329, aka, taprenepag isopropyl) (Figure 3) for the treatment of open-angle glaucoma and ocular hypertension (www.clinicaltrials.gov). In light of these findings, it is reasonable and important to question whether an acute or chronic treatment of EP2 antagonist would compromise healthy bones and weaken them. To address this question, we recently conducted a study with EP2 antagonist TG11-77. Upon chronic dosing of (134 mg/kg/day free base) TG11-77·HCl to mice in drinking water for 28 days, the tibia and femur from hind limbs were analyzed for bone mass through diaphyseal scan and trabecular network through metaphyseal scan by microcomputed tomography (μCT).^58^ Overall, this study showed that EP2 antagonist treatment has no adverse effect on bone volume and density in healthy mice. These results dampen the potential threat to healthy bones and strengthen the advancement of EP2 antagonist for clinical use.

## Opportunities

Inflammation is an ongoing feature found in several central nervous system and peripheral diseases. In general, inflammation affects >100 million people in the USA. The global anti-inflammatory market is projected to reach $135 billion by 2027 with 4.8% compound annual growth rate (CAGR). Nonsteroidal anti-inflammatory drugs (NSAIDs) and cyclooxygenase-2 (COX2) inhibitors looked promising, but they were limited by their gastrointestinal and cardiovascular toxicity. In many inflammatory conditions, induction of COX2 and mPGES-1 was observed, together leading to synthesis of PGE_2_ driving downstream signaling through EP2 and EP4 receptors by synthesis of cAMP, EP1 by immobilization of intracellular Ca^2+^, or EP3 via inhibiting cAMP. Among these four PGE2 receptors, EP2 seems to act as an inflammatory mediator in the majority of in vivo studies examined so far (see Strengths section), whereas EP4 can act as a proinflammatory or an anti-inflammatory agent depending on the disease context. For example, EP4 receptor acts as a proinflammatory agent in rheumatoid and osteoarthritis conditions^66,67^ but as an anti-inflammatory agent in cardiovascular and Alzheimer’s disease models.^68,69^ The role of EP1 and EP3 seems limited in terms of promoting inflammation. Therefore, EP2 provides a tremendous opportunity to develop targeted therapeutics that should bypass the adverse cardiovascular events found with the use of COX2 inhibitors rofecoxib (Vioxx) and valdecoxib (Bextra).^70,71^

Similarly, cancer impacts 18 million people worldwide, leading to about 10 million deaths a year. The global oncology market was US $286 billion in the year 2021, which is expected to reach $581 billion by 2030, with CAGR 8.2% from 2022 to 2030. There are many cancer subtypes impacting various segments of the population. The proliferation, tumor growth, and metastasis of many of these cancers is associated with the inflammatory tumor microenvironment. Interestingly, COX2, PGE_2_ and EP2 all are driving this malignant tumor growth; therefore, selectively targeting EP2 receptor should offer therapeutic advantages that are not found with the use of generic COX2 inhibitors and drugs with other mechanisms of action. One expects that targeting the EP2 receptor selectively downstream of complex signaling by COX2 should spare the physiologically relevant cardioprotective prostanoid receptor IP, which is activated by COX2 derived PGI_2_, and platelet modulator TP receptor, which is activated by COX2 derived TXA_2_ ligand.^72,73^

There are several medically unaddressed diseases for which treatments are urgently needed. Capturing the impacts and unmet needs of each disease is beyond the scope of this Perspective. Just to give an example, Alzheimer’s disease (AD), characterized by the onset of cognitive impairment, is the most common cause of dementia. It affects 6 million people in the USA, and this number is expected to grow to 14 million by 2050. According to a recent review (by Kim et al.),^74^ there were about 543 interventional clinical trials among the total of 2695 clinical trials conducted for AD between 2004 and 2021. Among these, 41% failed in phase III trials and 59% failed in Phase II. These trials included 64% disease modifying and 36% symptomatic agents. Nonetheless, the FDA approved a monoclonal antibody (drug) in 2021 (Biogen/Esai’s aducanumab; aka., Aduhelm), despite unanimous recommendations by the scientific review committee to reject the approval.^74^ This year (January 6, 2023), another antibody named lecanemab-irmb (Leqembi) was approved through the accelerated approval pathway by the FDA for the treatment of AD (www.leqembi.com). Due to the paucity of success in drug discovery and development against AD, exploring novel proof-of-concept drugs that work through a novel biological target such as EP2 receptor seems an important task for investigation. Moreover, due to known adverse cardiovascular events with chronic use of COX2 drugs,^70−73^ there is little to no incentive or enthusiasm to conduct additional long-term clinical trials with COX2 drugs for debilitating diseases such as AD,^75^ post-traumatic epilepsy, or other chronic neurodegenerative diseases. Thus, targeting EP2 receptors with small molecules provides enormous opportunities for clinical development.

## Potential Threats and Limitations for Targeting EP2 and Shortfalls with the Available Small Molecule Modulators

The real threats for targeting EP2 receptor by a pharmacological approach are elusive except for use against endometriosis. EP2 expression is strong in luminal epithelium at the implantation sites and may serve as a marker for uterine receptivity suggesting its role in embryo implantation in mouse and rat.^76,77^ Because endometriosis impacts women at childbearing age, this potential threat must be addressed with pharmacological antagonism with a specific EP2 antagonist, because EP4 and IP receptors are also highly expressed at the sites of embryo implantation in uterus, and they may play a compensatory role for EP2 in this context.

EP2 promotes cellular signaling cascades via several intracellular molecules and pathways. As discussed above, it mediates Gα_s_-dependent cAMP driven PKA and Epac signaling cascades on one side, which drive inflammation, neurodegeneration, and neuronal plasticity, and G-protein independent signaling via β-arrestin signaling on the other that drives cancer proliferation and metastasis and tumor development. Moreover, the anabolic activity of EP2 in the bone and bone marrow is also coupled to cAMP mediated signaling. All of these could give mixed conclusions to drug discovery and pharmaceutical communities and limited clarity on the therapeutic indication for which the advancement of EP2 drugs could be prioritized.

So far, there is one EP2 targeted drug, omidenepag isopropyl (aka, Omlonti) (Figure 3), is clinically approved by the FDA for the reduction of elevated intraocular pressure in patients with primary open-angle glaucoma or ocular hypertension (https://www.omlonti.com). A trailing second candidate in the class, PF-0417329 (prodrug of CP-544,326, see Figure 3), also underwent clinical evaluation in humans (NCT00934089) and it significantly reduced intraocular pressure in primary open-angle glaucoma and ocular hypertension (NCT00572455).^78^

As to the EP2 antagonists, one candidate EP2 antagonist, PF-04418948, went through Phase 1 human clinical trials examining the safety and tolerability of the compound by single and escalating doses (https://beta.clinicaltrials.gov/study/NCT01002963). The data seem compelling and showed dose-linear increase in AUC from 30 mg/kg to 1000 mg/kg (but not beyond) doses, and treatment was well tolerated with no cardiovascular events or renal toxicity (measured by KIM-1 molecule); however, it showed mild hyperbilirubinemia (dose-dependent increase in bilirubin) which is associated with its strong inhibition activity against blood transporter OATP1B1.^79^ Since then, Pfizer has made some organizational changes, and as a result the subsequent development of this project has been terminated (personal communication). Nonetheless, PF-04418948 is very selective to the EP2 receptor, and it was able to reverse the PGE_2_ induced relaxation of mouse trachea at IC_50_ = 2.7 nM; it suppressed butaprost induced cutaneous blood flow by oral-dosing at 3 mg/kg in rat.^32^ PF-04418948 is a carboxylic acid derivative and displayed low volume of distribution and clearance with terminal plasma half-life of 8.8 h with oral bioavailability of 78%;^32^ however, it is brain-impermeable, and therefore, it can be used for blocking peripheral EP2 effects. Likewise, Amgen has investigated EP2 as a target for drug discovery and identified a lead candidate from high-throughput screening (HTS) and SAR studies. The lead candidate molecule C52 seems to be a selective antagonist of EP2 over other prostanoid receptors, and it is highly brain-permeable (B/P ratio 0.7–0.9) with a plasma half-life of 3.4 h and oral bioavailability of 44%.^12^ However, Amgen did not pursue this project further for strategic reasons, and they closed the research site where this program evolved, and the program was also terminated (personal communications).

Our laboratory has made significant contributions in the creation and development of a novel class of EP2 antagonists. The first-generation research lead compound in the class is TG6-10-1,^14^ which has some structural weaknesses. It possesses an acryl amide moiety, which potentially acts as a Michael acceptor for a variety of proteins and amino acids to form adducts in biological systems, which could pose some limitations for clinical development. The second research lead candidate in the program was TG8-260, which is highly potent and orally bioavailable but is not brain-permeable (B/P ratio 0.04). Moreover, it has shown very potent cytochrome P450 (CYP) inhibition activity against several CYP450 enzymes;^51^ therefore, it has a potential limitation of displaying drug–drug interactions. Very recently, we have reported the preclinical characterization of current lead molecule TG11-77, which has passed several IND-related ADME-PK tests.^21^ In comparison to Pfizer compound PF-04418948 and Amgen compound C52, it has shown weak inhibition activity against blood transporters (unpublished), and it is currently going through additional pre-IND requisite dose–response toxicokinetic tests in dogs.

## Summary and Outlook

So far, clinical proof-of-concept (POC) with EP2 agonist omidenepag isopropyl was achieved for ophthalmic use to conclude that EP2 is a druggable target. However, clinical POC with an EP2 antagonist is yet to be achieved for any indication. Nonetheless, based on the substantial in vivo efficacy data from SE models in our laboratory, where EP2 antagonism was proven to be anti-inflammatory in three chemically induced models of SE (pilocarpine, kainate, diisopropyl-fluorophosphate (DFP)) in two rodent species (Table 2) and where the anti-inflammatory effect of EP2 antagonism has been translated into cognitive/memory improvements in those models, it is important to advance a clinical candidate toward attenuation or delay of the cognitive impairments in SE patients, and other patients (such as those suffering postoperative surgery or severe infections by RSV and SARS viruses) that are prone to develop cognitive impairments and also patients with autoimmune disorders like multiple sclerosis. Depending on the disease, the EP2 antagonist can be administered as a first line monotherapy (for cancer and other peripheral inflammatory diseases) or an adjunctive therapy along with first line antiseizure drugs for the SE indication. For the treatment of AD, it is crucial to identify the right time to begin the treatment and the duration of the treatment with a novel anti-inflammatory agent. These studies must be guided by the current understanding and the trajectory of microglial activation and its house-keeping performance (Aβ-clearance) during the course of development of Alzheimer’s disease.^80^ For the treatment of brain cancers, the studies must develop a brain-permeable candidate with requisite pharmacokinetics and acceptable ADMET properties that facilitate Q.I.D. or B.I.D. dosing in patients. These goals are all achievable in the foreseeable feature.

**Table 2:** Efficacy Markers in Mouse and Rat Models of SE with Emory EP2 Antagonists^a^

	TG6-10-1 (5 or 10 mg/kg)				
efficacy marker	in pilocarpine mouse SE	in DFP-rat SE	in kainite mouse SE	TG8-260 (25 mg/kg) in pilocarpine rat SE	TG11-77 (8.8 mg/kg) in pilocarpine mouse SE
delayed mortality	blunted	blunted	trend	no	yes
delayed weight	accelerated	accelerated	accelerated	no	no
neurologic function (nest building)	accelerated	b	accelerated	b	no
cytokine burst in the hippocampus	blunted	blunted	blunted	blunted	blunted
gliosis in the hippocampus	blunted	blunted	blunted	blunted	blunted
neurodegeneration in the hippocampus	blunted	blunted	blunted	no	no effect
memory deficit	b	blunted	b	b	blunted

a In the efficacy studies, rodents were exposed to listed EP2 antagonists for a short period of time, 2–30 h, following 1 h SE. TG8-260 is a brain-impermeable compound, whereas TG11-77 is brain permeable, and TG6-10-1 has excellent brain-permeability. Details are provided in Table 1 and the references cited in Table 1. Structures are shown in Figure 2.

b Not determined.

### Significance

- The EP2 receptor plays “yin–yang” physiological and pathological roles.
- The advent of selective EP2 antagonists and agonists contributed significantly to the conclusion that EP2 promotes inflammation in CNS diseases and cancer.
- With the FDA approval of omidenepag for glaucoma and sound beneficial effects of EP2 antagonism in status epilepticus and cancer models, the EP2 receptor seems to be a novel druggable target.
- This Perspective summarizes the pros and cons of targeting EP2 receptors with pharmacological agents.
